# Loss of T-Cell Multifunctionality and TCR-Vβ Repertoire Against Epstein-Barr Virus Is Associated With Worse Prognosis and Clinical Parameters in HIV^+^ Patients

**DOI:** 10.3389/fimmu.2018.02291

**Published:** 2018-10-04

**Authors:** Diana M. Hernández, Sandra Valderrama, Sandra Gualtero, Catalina Hernández, Marcos López, Maria Victoria Herrera, Julio Solano, Susana Fiorentino, Sandra Quijano

**Affiliations:** ^1^Grupo de Inmunobiología y Biología Celular, Departamento de Microbiología, Facultad de Ciencias, Pontificia Universidad Javeriana, Bogotá, Colombia; ^2^Grupo de Investigación en Enfermedades Infecciosas, Hospital Universitario San Ignacio, Facultad de Medicina, Pontificia Universidad Javeriana, Bogotá, Colombia; ^3^Grupo de Investigación Biomédica Traslacional, Fundación Cardiovascular de Colombia, Floridablanca, Colombia; ^4^Servicio de Hematología Hospital Universitario San Ignacio, Bogotá, Colombia

**Keywords:** HIV, EBV, non-Hodgkin B-cell lymphoma, memory T cells, effector T cells, T-cell multifunctionality, TCR-Vβ repertoire

## Abstract

Epstein-Barr virus (EBV) is an oncogenic virus associated with the development of aggressive and poor-prognosis B-cell lymphomas in patients infected with human immunodeficiency virus (HIV^+^ patients). The most important risk factors for these malignancies include immune dysfunction, chronic immune activation, and loss of T-cell receptor (TCR) repertoire. The combination of all these factors can favor the reactivation of EBV, malignant cell transformation, and clinical progression toward B-cell lymphomas. The overarching aim of this study was to evaluate the frequency, phenotype, functionality, and distribution of TCR clonotypes for EBV-specific T-cell subpopulations in HIV^+^ patients at different clinical stages and for HIV^+^ patients with B-cell lymphoma, as well as to establish their association with clinical variables of prognostic value. Factors were studied in 56 HIV^+^ patients at different clinical stages and in six HIV+ subjects with diagnosed B-cell lymphoma. We found a significant decrease in all subpopulations of EBV-specific CD4^+^ T cells from HIV^+^ patients at stage 3 and with B-cell lymphoma. EBV-specific effector CD8^+^ T cells, particularly effector memory cells, were also reduced in HIV^+^ patients with B-cell lymphoma. Interestingly, these cells were unable to produce IFN-γ and lacked multifunctionality in HIV+ patients. The TCR-Vβ repertoire, which is key for protection against EBV in healthy individuals, was less diverse in HIV^+^ patients due to a lower frequency of TCR-Vβ2^+^, Vβ4^+^, Vβ7.1^+^, Vβ9^+^, Vβ13.6^+^, Vβ14^+^, Vβ17^+^, Vβ22^+^ CD4^+^, Vβ14^+^, and Vβ17^+^ CD8^+^ T cells. HIV^+^ patients with positive plasma EBV loads (EBV^+^HIV^+^) had a noteworthy decrease in the levels of both TNF-α^+^ and multifunctional TNF-α^+^/IL-2^+^ and TNF-α^+^/IFN-γ^+^ CD8^+^ T cells. Altogether, our findings demonstrate that HIV^+^ patients have significant alterations in the immune response to EBV (poor-quality immunity) that can favor viral reactivation, escalating the risk for developing EBV-associated B-cell lymphomas.

## Introduction

HIV infection is characterized by the progressive depletion of CD4^+^ T-cell subpopulations (1, 2), the establishment of chronic, persistent immune activation (3), and premature immunosenescence (4). Alterations in the T-cell antigen receptor (TCR) repertoire have also been described in HIV^+^ patients by spectratyping, which involves evaluating the length distribution of the third complementary-determining region (CDR3) within the variable region of the β chain of the TCR (TCR-Vβ). In general, these studies showed deletions and expansions of some TCR-Vβ families (5, 6). These findings have demonstrated that HIV infection differentially modulates the frequencies of some T-cell clones, giving rise to a severely restricted TCR repertoire during chronic infection (6). In this way, HIV infection is responsible for impaired immune surveillance against infectious agents, which promotes the development of diseases associated with persistent and oncogenic pathogens, such as the opportunistic Esptein Barr Virus (EBV).

EBV is a ubiquitousγ-herpesvirus that infects more than 95% of the population worldwide. It is responsible for an asymptomatic, lifelong infection due to its interaction with B-cells, giving rise to the latent infection of memory B-cell pools in healthy adults (7). EBV is etiologically related to a wide range of human tumors, including B-cell lymphomas. A differential viral oncogenic protein profile is expressed during different stages of the latent phase (8) in tumor cells of different tissues (9, 10). These viral proteins contribute to tumor pathogenesis through the activation of different cell signaling pathways that, in turn, alter cell cycle, apoptosis and cell differentiation, influencing differential mechanisms of migration and survival in tumor cells (11, 12).

Previously, we reported that, in contrast with healthy individuals, patients with diffuse large B-cell lymphoma (DLBCL) had a less diverse TCR-Vβ repertoire, with deletion of the Vβ families that are relevant for the EBV-specific immune response (13). Notably, HIV^+^ patients with positive EBV plasma loads (EBV^+^HIV^+^) and viral genomes in tumors (EBER-1^+^ and EBER-2^+^ RNA transcripts) had a lower number of EBV-specific multifunctional CD8^+^ T cells. These findings established that chronic exposure to EBV during the course of infection and before the diagnosis of the malignancy could favor the expansion of EBV-infected B cell clones and the development of aggressive B-cell lymphomas with a high mortality rate (13).

Compared to non-infected individuals, HIV^+^ patients have a 60–200 times higher risk of developing B-cell non-Hodgkin lymphomas (14), and a high proportion of these cases are associated with active EBV infections (3). Several studies have evaluated the EBV-specific immune response in these patients using peptides derived from the EBV lytic and latent proteins (15). Importantly, during HIV infection, a progressive reduction in the number of EBV-latent protein (BZLF1 and BMLF)-specific IFN-γ^+^ CD8^+^ T cells is observed. On the other hand, HIV^+^ patients who have developed DLBCL have been found to display a significant depletion of EBV-specific functional CD8^+^ T cells, and this finding has been associated with increased plasma EBV loads (16). Accordingly, it is clinically pertinent to characterize the magnitude, quality and diversity of EBV-specific immune responses in HIV^+^ individuals because immunodeficiency (17, 18), the chronic stimulation of the immune system, and the exhaustion of lymphocyte subsets could trigger EBV reactivation and increase the risk of developing poor prognosis B-cell lymphomas.

Here, in this context, we assessed the TCR-Vβ repertoire and functionality of EBV-specific CD4^+^ and CD8^+^ T cell subpopulations from HIV^+^ patients in different clinical stages in comparison with a group of healthy individuals. Peripheral blood cells were stimulated with a complete EBV lysate (B95.8 cell line) and assessed by flow cytometry. We found that EBV^+^HIV^+^ patients at advanced clinical stages had a qualitatively impaired response to EBV, characterized by a lack of multifunctionality and decreased or deleted TCR-Vβ families that are important for the control of EBV in healthy individuals. These results suggest that EBV-specific immune responses in these patients are poorly protective and are quantitatively and qualitatively deficient. Overall, our findings demonstrate that these special immunological conditions in EBV+HIV+ patients are sufficient to trigger EBV reactivation in B-cell clones, its capabilities to transform cells, and the risk of evolution to B-cell lymphoma.

## Materials and methods

### Subjects and samples

Heparinized peripheral blood (PB) samples were collected from 27 EBV-seropositive healthy individuals (9 male and 18 female) with a median age of 41 years old (ranging from 22 to 83 years) and 62 EBV-seropositive HIV^+^ patients (55 male and 7 female) with a median age of 40 years old (ranging from 18 to 74 years) enrolled in the Infectology Program at Hospital Universitario San Ignacio (HUSI) in Bogota, Colombia. Relevant clinical characteristics of patients are summarized in Table 1. Sixteen patients were at stage 1, 20 were at stage 2 and 20 were at stage 3; 6 of 62 patients had B-cell non-Hodgkin lymphoma (19). This study was approved by the Research and Ethics Committee of the Faculty of Sciences of the Pontificia Universidad Javeriana (May 8th, 2014 session) and the Committee of Research and Ethics of the Faculty of Medicine of the Hospital Universitario San Ignacio (Ref. 2016–08). All subjects gave written informed consent in accordance with the Declaration of Helsinki.

**Table 1:** Clinical and biological features of HIV^+^ patients at different clinical stages of disease (*n* = 62).

	**Stage 1**	**Stage 2**	**Stage 3**	**Lymphoma**	***P*-value**
Sex	*n* = 16 Male: 16 (100%)	*n* = 20 Female: 5 (25%) Male: 15 (75%)	*n* = 20 Female: 1 (5%) Male: 19 (95%)	*n* = 6 Female: 1 (16.7%) Male: 5 (83.3%)	NS
Age	*n* = 16 Median: 31 (23–62 yr.)	*n* = 20 Median: 39 (23–74 yr.)	*n* = 20 Median: 41.5 (18–60 yr.)	*n* = 6 Median: 56 (48–67 yr.)	*P* < 0.05
Time of diagnosis (years)	*n* = 16 Median: 1.5 (0–11 yr.)	*n* = 20 Median: 4.5 (0−22 yr.)	*n* = 20 Median: 8 (1–19 yr.)	*n* = 5 Median: 7 (0–12 yr.)	*P* < 0.05
Leukocyte count/μL	*n* = 16 Median: 8152 (4,475–11,900)	*n* = 20 Median: 6100 (4,000–9,168)	*n* = 20 Median: 5450 (2,500–10,400)	*n* = 6 Median: 5192.5 (2,500–9,700)	*P* < 0.05
CD4^+^ T–cell/μL at diagnosis	*n* = 16 Median: 628 (507–1066)	*n* = 16 Median: 420,5 (243–605)	*n* = 20 Median: 178 (21–477)	*n* = 5 Median: 63 (13–206)	< 0.0001
CD4^+^ T-cell/μL at enrollment in the study	*n* = 16 Median: 672.5 (523–1,066)	*n* = 20 Median: 481 (246–877)	*n* = 20 Median: 396.5 (128–744)	*n* = 6 Median: 97 (13–379)	< 0.0001
CD8^+^ T-cell/μL at enrollment in the study	*n* = 16 Median: 846 (432–2,268)	*n* = 20 Median: 673.5 (261–1,114)	*n* = 20 Median: 682.5 (135–1,425)	*n* = 5 Median: 561 (242–791)	*P* < 0.05
Last HIV Load	*n* = 16 Detectable: 7 (43.8%) Undetectable: 9 (56.3%)	*n* = 20 Detectable: 6 (30%) Undetectable: 14 (70%)	*n* = 19 Detectable: 3 (15.8%) Undetectable: 16 (84.2%)	*n* = 6 Detectable: 3 (50%) Undetectable: 3 (50%)	NS
Antiretroviral therapy (ART)	*n* = 16 Yes: 13 (81.3%) No: 3 (18.8%)	*n* = 20 Yes: 17 (85%) No: 3 (15%)	*n* = 20 Yes: 20 (100%)	*n* = 5 Yes: 4 (80%) No: 1 (20%)	NS
HAART adherence (%)	*n* = 13 Median:100% (0–100)	*n* = 17 Median:100% (0–100)	*n* = 20 Median:100% (0–100)	*n* = 3 Median:100% (0–100)	NS
VF	*n* = 16 No: 16 (100%)	*n* = 19 Yes: 4 (21.1%%) No: 15 (78.9%)	*n* = 20 Yes: 8 (40%) No: 12 (60%)	*n* = 5 Yes: 1 (20%) No: 4 (80%)	*p* = 0.015
IF	*n* = 16 No: 16 (100%)	*n* = 20 No: 20 (100%)	*n* = 20 Yes: 2 (10%) No: 18 (90%)	*n* = 5 Yes: 3 (60%) No: 2 40(%)	*p =* 0.002
Co- infections	*n* = 16 Yes: 10 (62.5%) No: 6 (37.5%)	*n* = 20 Yes: 12 (60%) No: 8 (40%)	*n* = 20 Yes: 16 (80%) No: 4 (20%)	*n* = 6 Yes: 6 (100%)	NS
Comorbidities (%)	*n* = 16 Yes: 3 (18.8%) No: 13 (81.3%)	*n* = 20 Yes: 5 (25%) No: 15 (75%)	*n* = 20 Yes: 8 (40%) No: 12 (60%)	*n* = 5 Yes: 2 (40%) No: 3 (60%)	NS
AIDS-defining diseases	*n* = 16 No: 16 (100%)	*n* = 20 No: 20 (100%)	*n* = 20 Yes: 15 (75%) No: 5 (25%)	*n* = 5 Yes: 3 (50%) No: 3 (50%)	(*p* < 0.001)
EBV Load	*n* = 16 Pos: 4 (25%) Neg: 12 (75%)	*n* = 20 Pos: 2 (10%) Neg: 18 (90%)	*n* = 19 Pos: 1 (5.3%) Neg: 18 (94.7%)	*n* = 5 Pos: 3 (60%) Neg: 2 (40%)	NS
Anti-EBV VCA IgG antibodies	*n* = 2 IgG^+^: 2 (100%)	*n* = 12 IgG^+^: 12 (100%)	*n* = 15 IgG^+^: 15 (100%)	*n* = 2 IgG^+^: 2 (100%)	NS
Anti-EBV VCA IgM antibodies	*n* = 2 IgM^−^: 2 (100%)	*n* = 10 IgM^+^: 7 (70%) IgM^−^: 3 (30%)	*n* = 15 IgM^+^: 8 (53.3%) IgM^−^: 7 (46.7%)	*n* = 2 IgM^+^: 1 (50%) IgM^−^: 1 (50%)	

*Last viral load: detectable, >40 copies/mL; undetectable, < 40 copies/mL; VF, Virological failure; Plasma viral load (PVL) > 50 copies/mL after 24 weeks of ART; IF, Immunological failure, inability to obtain an adequate count of CD4^+^ T cells in spite of having a PVL < 50 copies/mL; ART, Antiretroviral therapy; AIDS, Acquired immunodeficiency syndrome; NS, non-significant*.

EBV seropositivity was determined by a semiquantitative ELISA for the detection of IgM and IgG antibodies against the viral capsid antigen (VCA) using the RIDAS SCREEN EBV VCA IgG and IgM kits (R-Biopharm AG. Darmstadt, Germany). Plasma EBV load was determined by real-time PCR (qPCR) for detection of the viral EBNA-1 coding gene sequence with the EBV LightMix® Kit from TIB MOLBIOL and a Light Cycler 2.0 from Roche Diagnostics (Roche, Bogotá, Colombia). Samples were analyzed within 12 h after collection.

### *In vitro* evaluation of EBV-specific T-cell responses

Total peripheral blood samples were stimulated in *in vitro* cultures with EBV lysate, as previously described (20). Briefly, a 750-μL aliquot of blood sample, diluted 1:1 with RPMI 1640, was treated with 5 μg/mL of EBV lysate (B95.8; Zeptometrix Corporation. Buffalo, NY), 1 μg/mL anti-CD28 mAb (clone L293; BD Biosciences, San Jose, CA), and 1 μg/mL anti-CD49d mAb (clone L25; BD Biosciences) for 6 h at 37°C in a 5% CO_2_ atmosphere. As a negative control, a 250-μL aliquot of diluted blood sample was cultured under the same conditions but without EBV lysate.

For evaluation of naïve, effector and memory T-cell subpopulations, cells were stained for 15 min with the following fluorochrome-conjugated anti-human mAbs: anti-CD3-PECy7 (clone SK7; BD Pharmingen, San Diego, CA), anti-CD4-PerCP (clone HP2/6; Immunostep SL, Salamanca, Spain), anti-CD8-APC (clone MEM-31; Immunostep SL), anti-CD45RA-FITC (clone GRT22; Immunostep SL), and anti-CCR7-PE (clone AB12; Immunostep SL). Afterwards, samples were lysed with 1X FACS Lysing solution (BD Biosciences) for 15 min in the dark at room temperature.

After washing twice, stained cells were measured in a FACSAria II Flow Cytometer using the FACSDiva software program (BD) using a two-step procedure. In the first step, 5 × 10^4^ events from the whole PB cellularity were measured, while in the second step, data of approximately 1–2 × 10^5^ CD3^+^ T-cells were specifically stored. The results were analyzed in terms of cell number/μl, taking into account the total leukocyte count from the hemograms.

### Detection of cytokines

EBV-stimulated and non-stimulated cultures were prepared as previously described. Intracellular cytokines were determined by adding 1 μg/mL Brefeldin A (BFA; BD Biosciences) to cultures at the second hour of the 6 h incubation period. As positive controls, 1 × 10^6^ leukocytes were cultured with a polyclonal stimulus (10 ng/mL PMA and 1 μg/mL ionomycin) for 4 h at 37°C and 5% CO_2_ atmosphere (13, 19). Then, cells were stained with the following fluorochrome-conjugated anti-human mAbs: anti-CD3-PECy7 (clone SK7; BD Pharmingen), anti-CD4-PerCP (clone HP2/6; Immunostep SL), and anti-CD8-APC-Cy7 (SK1; BD Pharmingen) for 15 min at room temperature and in the dark. Cells were washed, fixed and permeabilized using the Cytofix/Cytoperm™ Plus kit with Golgi Plug™ (BD Biosciences) containing BFA. Then, cells were stained with the following fluorochrome-conjugated anti-human mAbs: anti-IFN-γ-FITC (clone 45-15; Miltenyi Biotec, Bergisch Gladbach, Germany), anti-TNFα-PE (clone cA2; Miltenyi Biotec), and anti-IL2-APC (clone MQ1-17H12BD; BD Biosciences) for 30 min in the dark at room temperature.

Stained cells were analyzed in a FACSAria II Flow Cytometer (Becton Dickinson, San Jose, CA) using the FACSDiva software program (BD) with a two-step procedure as described in the previous section.

In a different set of experiments, blood samples were cultured and stimulated with EBV (6 h) or PMA + ionomycin (4 h) as previously described, but in the absence of BFA to allow for the secretion of cytokines. Then, supernatants were collected, and cytokines were quantified using the CBA Human Th1/Th2 cytokine kit II (BD Biosciences). This kit uses 6 types of fluorescent-linked beads to capture mAbs against human IL-2, IL-4, IL-6, IL-10, TNF-α, and IFN-γ. The detection limits of the kit are 2.6, 2.8, 3.0, and 7.1 pg/mL for IL-2/IL-4, TNF-α/IL-10, IL-6, and IFN-γ, respectively. Samples were acquired in a FACSAria II flow cytometer and analyzed with the FCAP Array software (BD Biosciences). Levels of cytokines were also measured in sera samples as described.

### Assessment of the TCR-Vβ family repertoire

Quantitative analysis of 24 TCR-Vβ families was carried out in basal (without stimulus) and EBV-stimulated conditions using the IOTest® β Mark TCR Vβ Repertoire Kit (Beckman Coulter, Marseille, France) as described elsewhere (20). Briefly, PB samples were stained with different combinations of three anti-human TCR-Vβ mAbs conjugated to FITC, PE, and FITC/PE (corresponding to 24 different specificities); the mAbs were anti-CD3-PECY7, anti-CD4-PerCP, and anti-CD8-APC (13). Stained samples were evaluated in a FACSAria II flow cytometer and analyzed with the FACSDiva v.6.1.3 software.

### Statistics

Non-parametric statistics were used for analyzing data. Quantitative variables (relative and absolute frequencies) were described as medians and ranges using SPSS Statistic 23 software (IBM). Differences between independent groups were evaluated with the Mann-Whitney *U*-test, and differences between paired groups were evaluated with the Wilcoxon and the sign tests.

The multifunctionality analysis was carried out using 10,000 permutations calculated with SPICE version 5.3 software. A *p*-value < 0.05 was considered significant.

## Results

### Clinical and biological data of HIV^+^ patients enrolled in the study

Clinical database analyses of HIV^+^ patients showed a higher proportion of men (55/62; 88.7%) than of women (7/62; 11.3%). HIV^+^ patients with lymphoma were significantly older (median = 56 years; range = 48–67) than were those without malignancy (*p* < 0.05). Overall, 13/60 (21.6%) patients presented with virological failure (VF; *p* = 0.015), 5/61 (8.19%) had immunological failure (IF; *p* = 0.002), and 18/62 (29.03%) had AIDS-defining diseases (*p* < 0.001). It is important to remark that patient features of worse prognosis were mainly at clinical stages 2 and 3, independent of the presence of lymphoma. Additionally, plasma EBV load (these correspond to the cases with active EBV infection) was detected in 10/60 (16.7%) HIV^+^ patients, with higher loads in 4/10 patients at stage 1. On the other hand, anti-EBV (VCA) IgG antibodies results were positive in 31/31 patients analyzed (Table 1). It is important to note that all healthy controls were seropositive for EBV (past infection) and did not have active infection (determined by viral load analysis).

### HIV^+^ patients at stage 3 lack EBV-specific multifunctional T cells regardless of the presence of lymphoma

**Figure 1:**
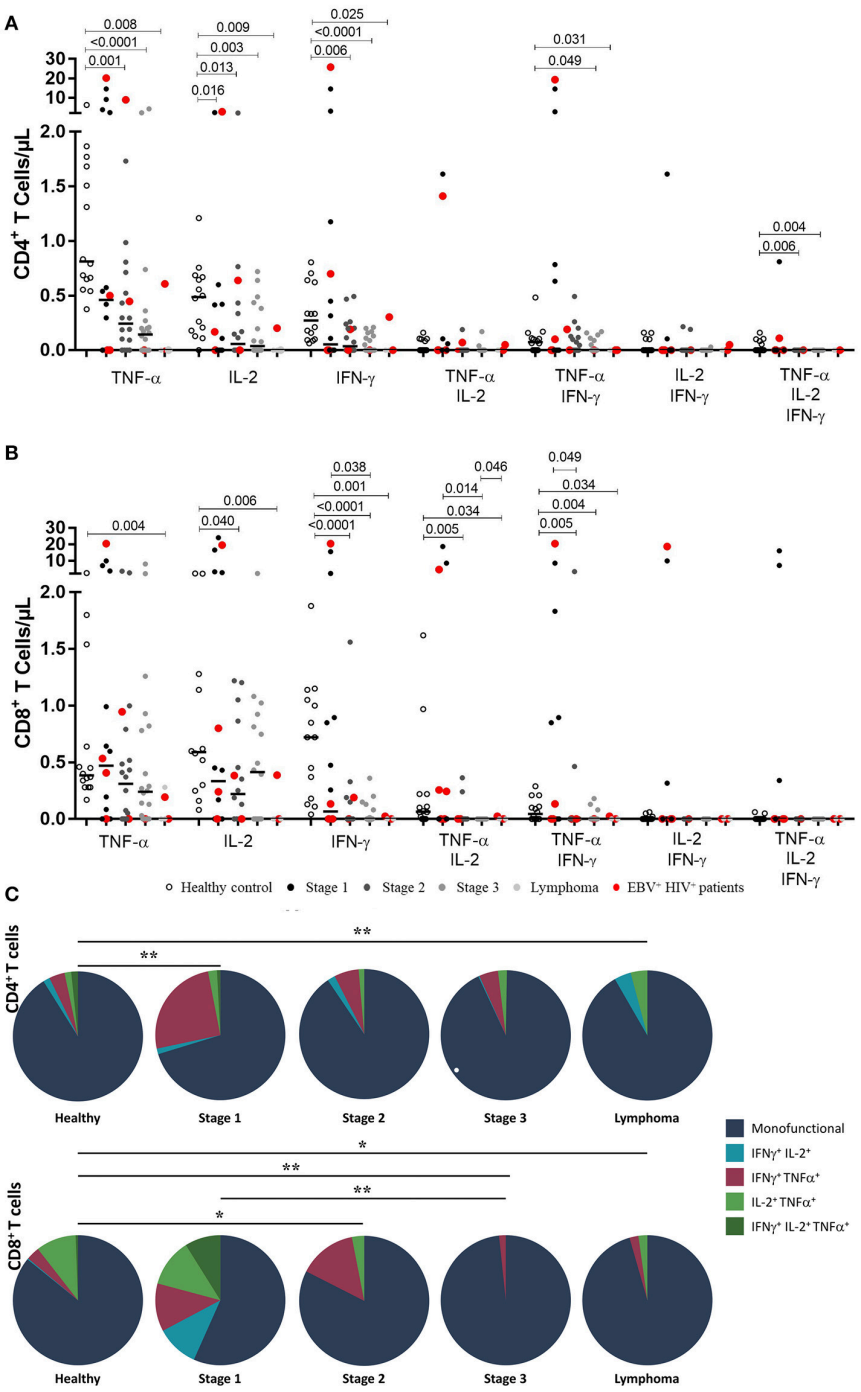
Functional EBV-specific T cell response in HIV^+^ patients. Peripheral blood samples of HIV^+^ patients at different stages of disease and healthy controls were cultured *in vitro* with EBV lysate. Counts of mono- and multifunctional (TNF-α^+^, IFN-γ and/or IL-2) CD4^+^** (A)** and CD8^+^** (B)** T cells were analyzed by flow cytometry. Net (EBV minus basal) counts are shown. Bold lines represent median values. Mann-Whitney *U*-test was used for comparisons between groups. **(C)** CD4^+^ and CD8^+^ T cells that coexpress intracellular cytokines after stimulation with EBV are shown. The colors in the pie charts depict the coexpression of the cytokines: one (dark blue), two (light blue, red and light green) and three (dark green). The *p* values of the permutation test in the coexpression analysis are shown (*p < 0.05 and **p < 0.001).

**Figure 2:**
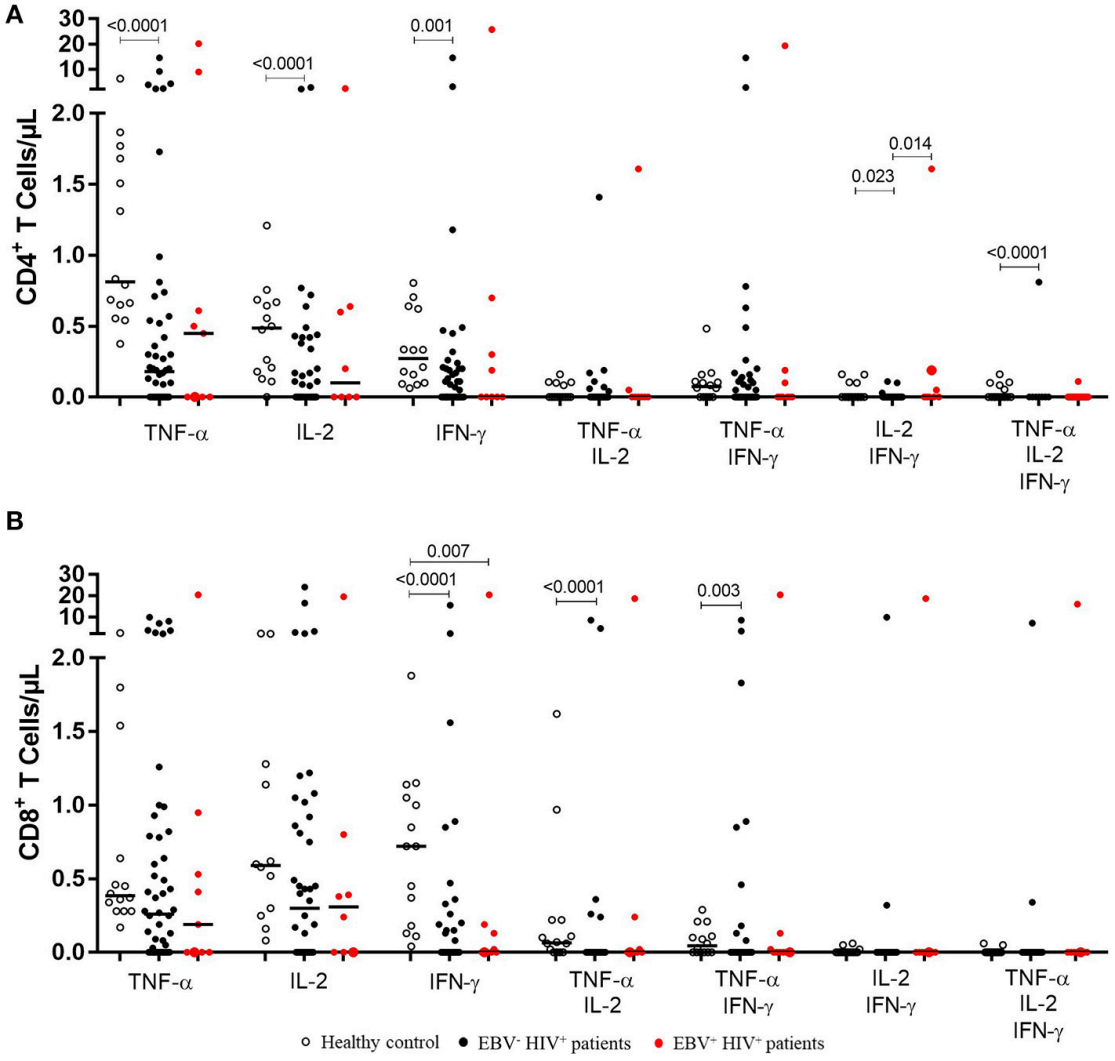
Functional EBV-specific T cell response in EBV^−^HIV^+^ vs. EBV^+^HIV^+^ patients. Peripheral blood samples of HIV^+^ patients and healthy controls were cultured *in vitro* with EBV lysate. Counts of mono- and multifunctional (TNF-α^+^, IFN-γ and/or IL-2) CD4^+^** (A)** and CD8^+^** (B)** T cells were analyzed by flow cytometry. Net (EBV minus basal) counts are shown. Bold lines represent median values. Mann-Whitney *U*-test was used for comparisons between groups.

In EBV-stimulated cultures, CD4^+^ T cells from HIV^+^ patients at stage 1 had lower numbers of IL-2-producing cells. This finding is a significant difference compared with that of healthy controls (*p* = 0.016). Monofunctional CD4^+^ T cells from patients at stages 2 and 3 were less abundant than those in healthy controls, as shown by TNF-α+ (stage 2, *p* = 0.001; stage 3, *p* < 0.001), IFN-γ^+^ (stage 2, p = 0.006; stage 3, *p* < 0.001); and IL-2^+^ (stage 2, *p* = 0.013; stage 3, *p* = 0.003) counts in Figure 2. On the other hand, patients with lymphoma exhibited a loss of functional EBV-specific CD4^+^ T cells, as demonstrated by TNF-α^+^ (*p* = 0.008); IFN-γ+ (*p* = 0.025); and IL-2^+^ (*p* = 0.009) counts. Likewise, a significant (*p* < 0.05) lack of multifunctional (TNF-α^+^/IFN-γ^+^/IL-2^+^) and TNF-α^+^/IFN-γ^+^ CD4^+^ T cell subsets was observed in more advanced stages of the disease (Figure 1A).

In the CD8^+^ T cell compartment, TNF-α^+^, IFN-γ^+^ and IL-2^+^ EBV-specific responses were gradually reduced in more advanced disease (*p* < 0.05). Importantly, this decrease was more critical in cases of lymphoma. Similarly, there was a gradual decrease in the number of T lymphocytes producing TNF-α^+^/ IL-2^+^ and TNF-α^+^/ IFN-γ^+^ with the progression of the disease (*p* < 0.05; Figure 1B).

The global analysis of the monofunctionality and multifunctionality is represented in Figure 1C, excluding the T lymphocytes that did not produce any cytokines. The figure shows that stage 1 patients still had low numbers of multifunctional cells and, particularly in 5 cases, higher numbers of IFN-γ+ /TNF-α+ T–cells were found. On the other hand, in more advanced stages of the disease, the loss of multifunctionality and the loss of IFN-γ+ /TNF-α+ T–lymphocytes were observed in cases of lymphoma (*p* < 0.05).

Bearing in mind that EBV has a significant effect on the role of the specific memory T lymphocytes controlling this chronic and persistent infection and that in our cohort 10 patients had active infection, we analyzed the differences of monofunctionality and multifunctionality between HIV^+^EBV^−^ and HIV^+^EBV^+^ cases. We also found that in EBV^+^HIV^+^ versus healthy controls, the response against EBV was characterized mainly by a reduction (*p* = 0.007) in the number of IFN-γ^+^ CD8^+^ T cells (Figure 2B). In CD4 + T lymphocytes, no statistically significant differences were found when comparing HIV^+^EBV^−^ vs. HIV^+^EBV^+^ cases (Figure 2A).

In general, these results mean that a lack of functional and specific T cells in HIV^+^ patients at advanced stages of disease (2, 3, and lymphoma) produces a low-quality immune response against EBV.

Cultures exposed to the polyclonal stimulus (PMA + ionomycin) showed that CD4^+^ T cells from HIV^+^ patients with lymphoma were not able to produce TNF-α, IFN-^γ^, and/or IL-2 (*p* < 0.05). Similarly, EBV^+^HIV^+^ patients had lower counts of IFN-^γ+^ CD4^+^ T cells in comparison with those of healthy controls (*p* = 0.035; data not shown). On the other hand, an increase in the frequencies of TNF-α^+^, IFN-^γ+^, and IL-2^+^ CD8^+^ T cells was observed in HIV^+^ patients at stage 1 (*p* < 0.01), 2 (*p* < 0.01), and 3 (*p* < 0.01) compared to those in healthy individuals. These results suggest a greater activation status of the immune response, with the exception of HIV^+^ patients with lymphoma, who exhibited lower counts of mono- and multifunctional CD8^+^ T cells in relation to those of healthy controls (*p* < 0.05). Likewise, CD8^+^ T cells from EBV^+^HIV^+^ patients showed a decreasing trend for multifunctionality (IFN-^γ+^/IL-2^+^) in comparison with those of healthy individuals (*p* = 0.064; data not shown). These findings demonstrate the significant loss of functionality in CD4^+^ and CD8^+^ T cells in response to non-specific/polyclonal stimuli, mainly in patients with lymphoma.

### Stage 3 HIV^+^ patients do not synthesize IFN-γ^+^ and IL-2^+^ in response to EBV, independently of the presence of lymphoma

**Figure 3:**
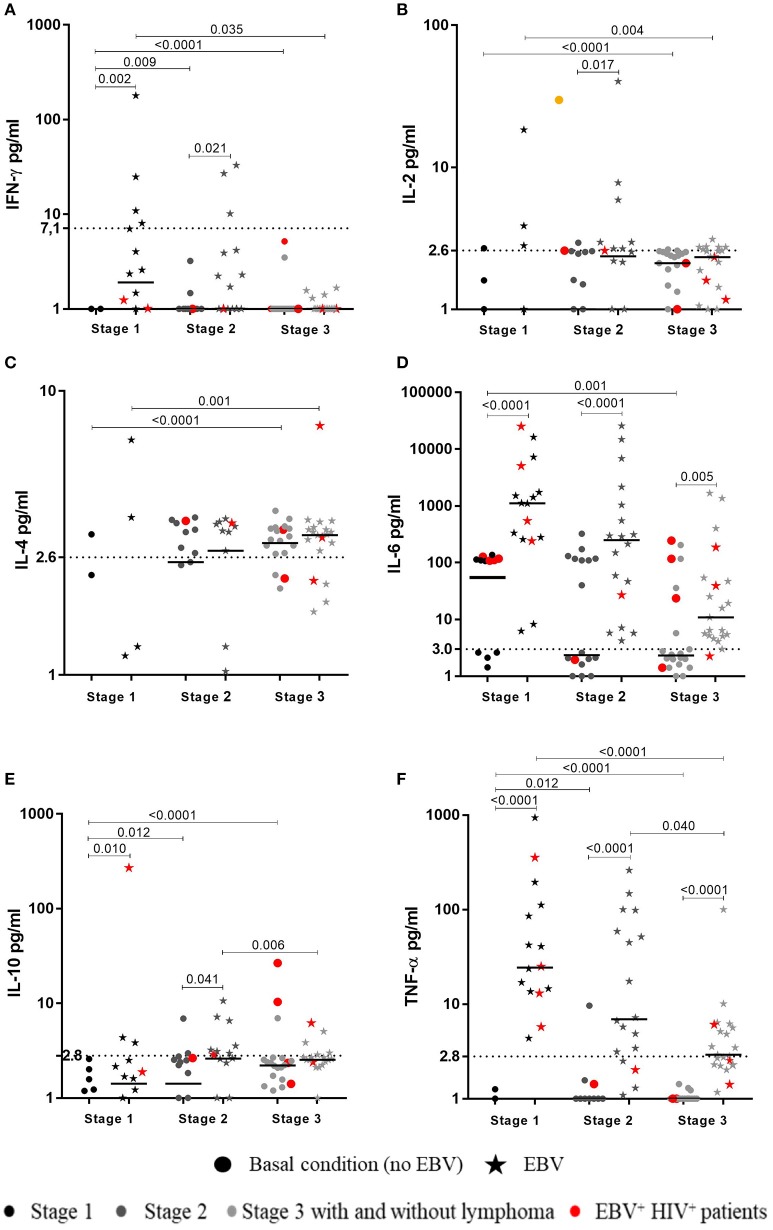
Cytokines synthesized in response to EBV by HIV^+^ patients. Peripheral blood samples of HIV^+^ patients at different stages of disease and healthy controls were cultured *in vitro* at basal (without EBV) and EBV-stimulated conditions without BFA. Supernatants were collected to measure the concentration (pg/mL) of cytokines by CBA and flow cytometry. The levels of IFN-γ **(A)**, IL-2 **(B)**, IL-4 **(C)**, IL-6 **(D)**, IL-10 **(E)** and TNF-α (**F**), are shown, respectively. Bold lines represent median values. Mann-Whitney *U*-test was used for comparisons between groups of individuals. Wilcoxon signed-rank Test was used for comparison between basal and EBV conditions. Dotted lines correspond to the limit of detection for every cytokine.

In cultures at basal conditions (without EBV stimuli), 23/56 (41%) HIV^+^ patients showed detectable levels of IL-6; 8/18 (44.7%) were at stage 2 and 7/22 (31.5%) at stage 3. Likewise, 7/9 (77.7%) EBV^+^HIV^+^ patients showed detectable levels of IL-6. In addition, 3/22 (13.5%) patients at stage 3 had cultures with assessable levels of IL-10. Two of the patients were EBV^+^ (Figure 3).

In response to EBV, detectable levels of IL-6, TNF-α, and IL-4 were found in 54/55 (98%), 42/55 (76%), and 26/55 (47%) HIV^+^ patients at all respective clinical stages (1–3). IL-6 was also detected in all EBV^+^HIV^+^ patients. However, concentrations of these cytokines in patients at stages 2 and 3, and in patients with lymphoma, were lower than those in healthy individuals (*p* < 0.01). In addition, cells of patients at stage 3 with lymphoma and cells of EBV^+^HIV^+^ individuals were not able to synthesize IFN-γ. Patients at stages 1 and 2 showed lower levels of cytokines than those in healthy controls (*p* < 0.05). IL-2 was detectable in cultures of 16/55 (29%) HIV^+^ patients, most of whom were in stage 2 (7/55) and stage 3 (6/55). IL-2 was not quantifiable in cultures of patients with lymphoma or EBV^+^HIV^+^, and IL-10 was only detectable in 15/55 (27%) patients.

It is important to note that higher levels of TNF-α and IL-6 in response to EBV were observed in cultures of all HIV^+^ patients in comparison with the basal values (without EBV). Similarly, the IFN-γ and IL-10 levels were also higher in patients at stage 1 (*p* < 0.01) and 2 (*p* < 0.05. Accordingly, the level of IL-2 was only higher in 7/18 (39%) patients at stage 2 (*p* = 0.017; Figure 3).

On the other hand, it must be emphasized that, after polyclonal (PMA + ionomycin) stimulus, IFN-γ was not detected in cultures of 88.8% HIV^+^ patients (*p* < 0.001). This cytokine was only detectable in cultures of 5/16 patients at stage 3, two of whom had lymphoma (data not shown). Finally, EBV^+^HIV^+^ patients presented lower numbers of IFN-γ+ subset (*p* = 0.001) than those of healthy individuals. The results reveal an important finding detected only in patients at advanced clinical stages (2 and 3 with and without lymphoma). These patients have a notable functionally reduced response to a non-specific stimulus, an outcome of their altered immunological and virological conditions (data not shown).

### HIV^+^ patients have a less diverse TCR-Vβ repertoire after stimulation with EBV

**Figure 4:**
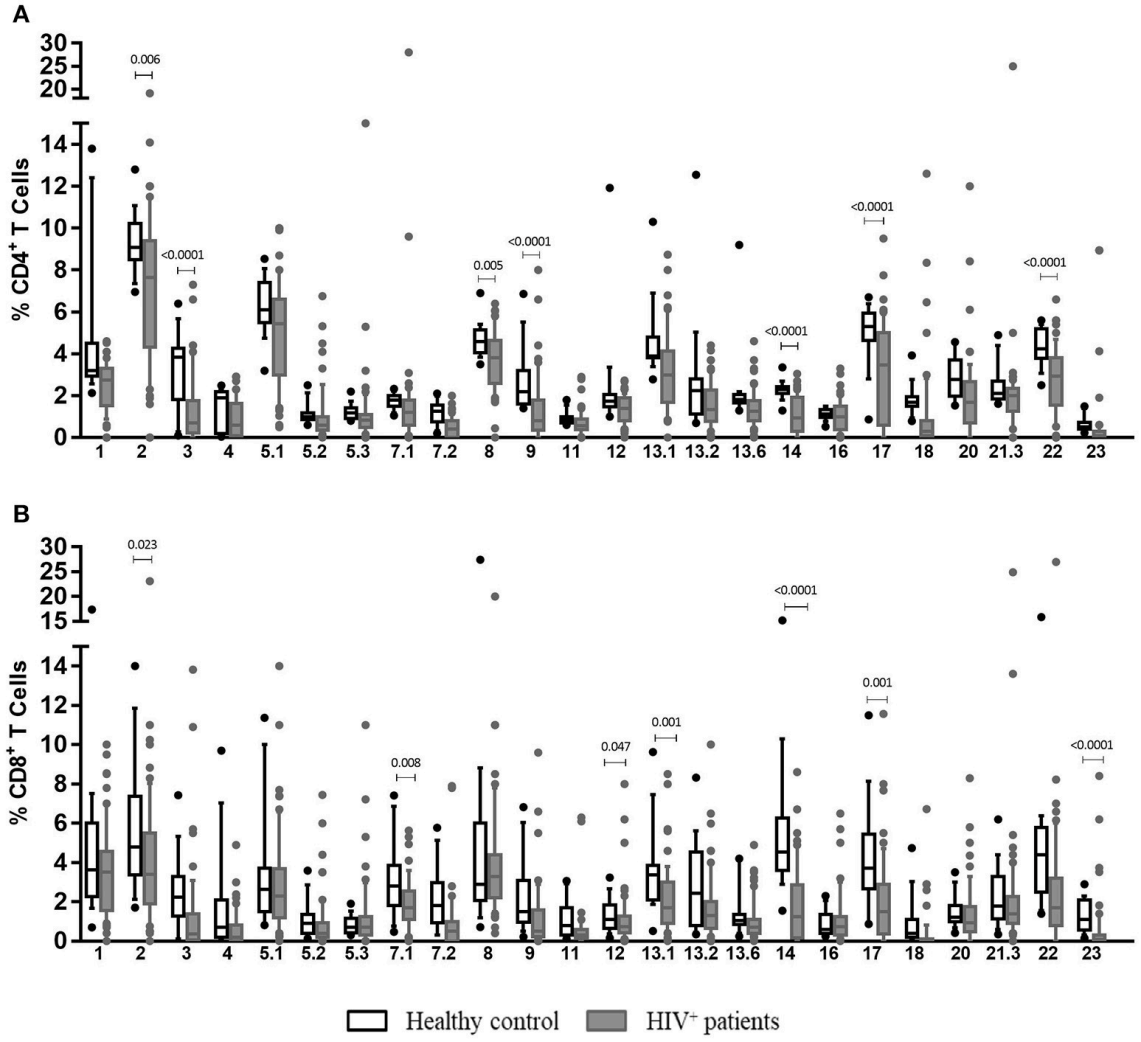
Clonotypic distribution of T cells from HIV^+^ patients at basal conditions. Peripheral blood samples of HIV^+^ patients and healthy controls were cultured *in vitro* at basal (without EBV) conditions. The distribution of CD4^+^** (A)** and CD8^+^** (B)** T cells positive for any of 24 TCR-Vβ families was analyzed with specific mAbs and flow cytometry. Box and whisker plots show range, median, and interquartile range of percentage of T cells positive for individual Vβ families. Mann-Whitney *U*-test was used for comparisons between groups.

The analysis of TCR-Vβ diversity was originated with an overall comparison of frequencies of CD4^+^ and CD8^+^ T cells expressing any of the 24 TCR**V**β families studied in HIV^+^ patients and healthy individuals at the basal level. As shown in Figure 4A, HIV^+^ patients had a lower frequency of TCR-Vβ3^+^, Vβ9^+^ and Vβ14^+^ (*p* < 0.001), Vβ2^+^ (*p* = 0.006), Vβ8^+^ (*p* = 0.005), Vβ17 (*p* < 0.001), and Vβ22^+^ (*p* < 0.001) CD4^+^ T cells. These are the most representative families in healthy individuals (19) (Figure 4A). Correspondingly, lower frequencies of the most representative families expressed in CD8^+^ T cells from healthy individuals were also observed in HIV^+^ patients: Vβ23^+^ (*p* < 0.001), Vβ2^+^ (*p* = 0.023), Vβ7.1+ (*p* = 0.008), Vβ13.1^+^ (*p* = 0.001), Vβ14^+^ (*p* < 0.001), and Vβ17^+^ (*p* = 0.001) (19) (Figure 4B). Altogether, the average of families detected in CD4^+^ and CD8^+^ T cells from HIV^+^ patients was 48.7% and 41.6 vs. 68.1% and 59.8% in healthy controls, respectively. These findings suggest that, in contrast to healthy controls, HIV^+^ patients displayed a more restricted TCR repertoire, with alterations in the frequency of some families that are important in the recognition of different pathogens. This effect, sequentially, could result in a decreased ability for protection in those patients.

Evaluation of TCR-Vβ repertoire in T cells from HIV^+^ patients at different stages allowed us to observe that alterations in some families could be characteristic of some clinical stages. A good example of this, for instance, is that Vβ1, Vβ2, Vβ5.3, Vβ12, and Vβ20 families were only observed at lower frequencies in patients at stage 3, independently of the presence of lymphoma (*p* < 0.05). In addition, Vβ5.1 (*p* = 0.039) and Vβ7.1 (*p* = 0.027) were exclusively detected in patients at stage 2 (*p* < 0.05), and the Vβ23 family frequency was significantly lower in patients at stages 1 and 3 (with or without lymphoma) than that in healthy individuals (*p* < 0.001; Figure S1A).

The frequency of Vβ3^+^ CD8^+^ T cells was significantly lower in patients at stage 2 and 3, irrespective of the presence of lymphoma, than that in healthy individuals (*p* < 0.001; Figure S1B). Additionally, Vβ7.1^+^ and Vβ2^+^ CD8^+^ T cells were only detected in patients at stage 2 (*p* = 0.007) and in stage 3 patients without lymphoma (*p* = 0.041). Lower frequencies of Vβ23^+^ CD8^+^ T cells were also observed in patients at stages 1 (*p* = 0.000) and 3 (*p* < 0.001), regardless of the presence of lymphoma and in patients with lymphoma (*p* = 0005). Finally, patients with lymphoma presented an overall significant reduction in the number of Vβ11^+^ CD8^+^ T cells (*p* = 0.007; Figure S1B).

**Figure 5:**
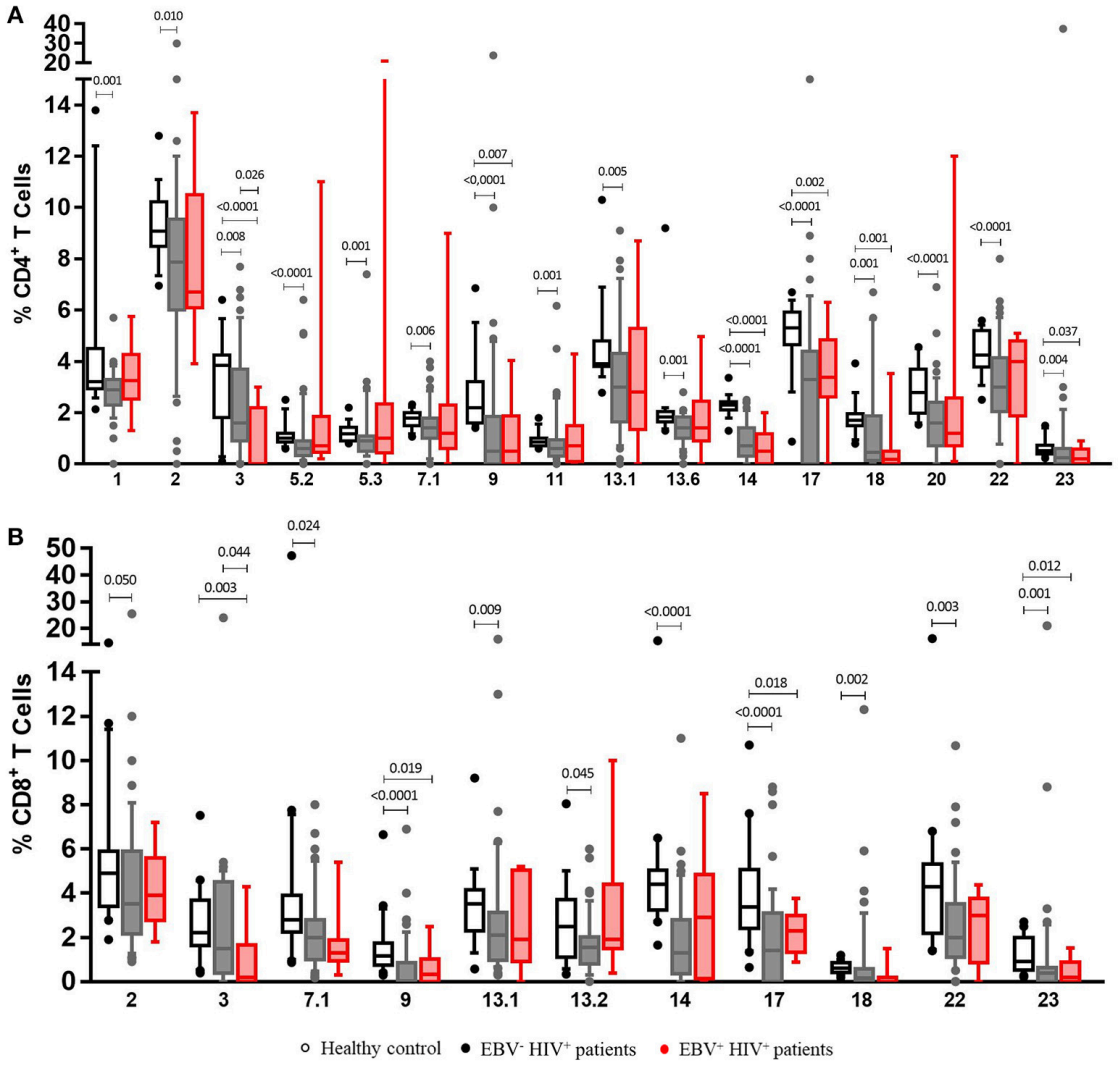
Clonotypic distribution of T cells comparing EBV^−^HIV^+^ versus EBV^+^HIV^+^ patients under EBV-stimulated conditions. Peripheral blood samples of HIV^+^ patients and healthy controls were cultured *in vitro* with EBV lysate. The distribution of CD4^+^** (A)** and CD8^+^** (B)** T cells positive for TCR-Vβ families was analyzed with specific mAbs and flow cytometry. Box and whisker plots show range, median, and interquartile range of percentage of T cells positive for individual Vβ families. Mann-Whitney *U*-test was used for comparisons between groups.

Although due to the experimental design, the specificity of the TCRVβ families against EBV cannot be demonstrated with accuracy, we found that after stimulation with EBV lysate, EBV^+^HIV^+^ patients had lower counts of Vβ3^+^ (*p* = 0.026) CD4^+^ T cells and Vβ3^+^ (*p* = 0.044) CD8^+^ T cells than did EBV^−^HIV^+^ patients (Figure 5). In addition, lower frequencies (*p* = 0.000) were also observed in Vβ9^+^, Vβ17^+^, and Vβ23^+^ CD4^+^, and CD8^+^ T cells in EBV^+^HIV^+^ and EBV^−^HIV^+^ patients in comparison with those in healthy individuals (Figure 5). These families have been shown to participate in EBV-specific immune responses in healthy individuals (19).

Overall, HIV^+^ patients showed a markedly impaired TCR-Vβ repertoire in CD4^+^ and CD8^+^ T cells. Moreover, deletions of CD4^+^ and CD8^+^ T cells positive for some TCR-Vβ families were also observed in HIV^+^ patients with lymphoma, in whom they can have a greater impact.

### Association of results from HIV^+^ EBV^+^ patients with clinical and biological parameters

Virological failure (VF), defined as a high HIV load caused by an unsuccessful antiretroviral therapy (ART), produces a reduction of naïve and central memory CD4^+^ T cells, which is attributed to the uncontrolled viremia. As shown in Figure S2A, such findings were observed in patients with VF in contrast with those who did not present that clinical condition (*p* < 0.05; Figure S2A). Likewise, CD8^+^ T_N_ cell counts were lower at basal (*p* = 0.031) and post-EBV-stimulus (*p* = 0.032) conditions in patients with VF.

**Figure 6:**
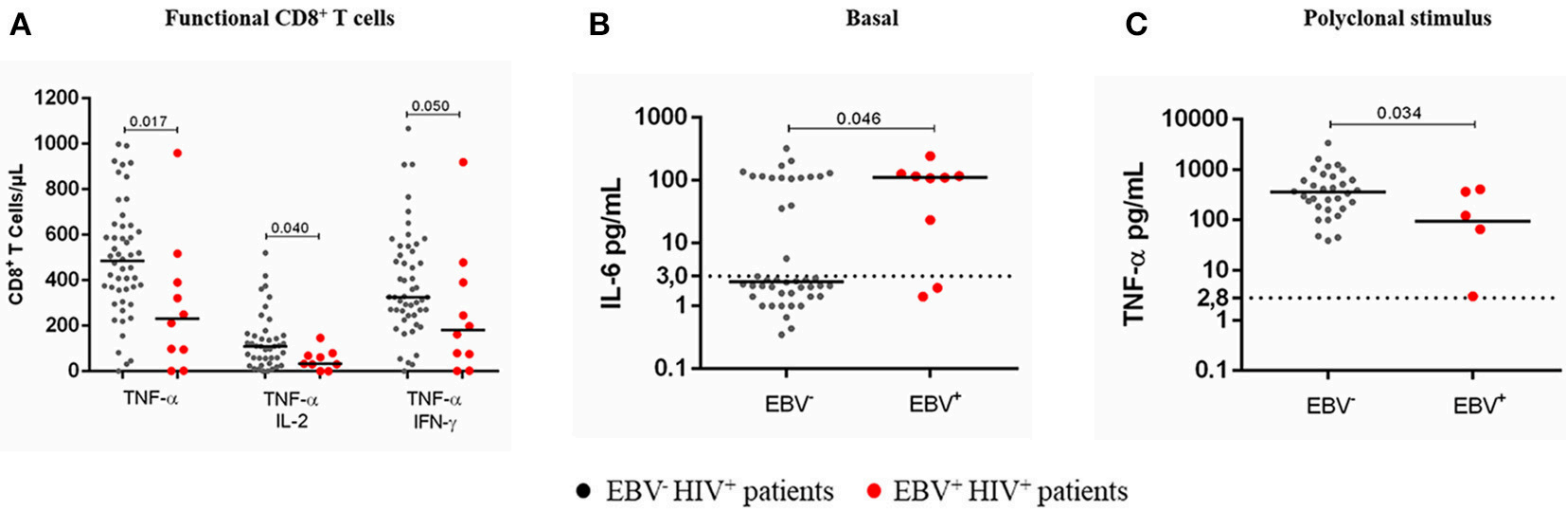
Cytokine response to a polyclonal stimulus in EBV^+^HIV^+^ patients. Peripheral blood samples of HIV^+^ patients were cultured *in vitro* at basal (without stimulus) or polyclonal (PMA + ionomycin)-stimulated conditions with BFA. Monofunctional and multifunctional (TNF-α^+^, IFN-γ^+^ and/ or IL-2^+^) T cells were characterized using mAbs and flow cytometry. The results for CD8^+^ T cells are shown **(A)**. Parallel cultures were made without BFA for collecting supernatants and measuring the concentration of soluble cytokines by CBA and flow cytometry. Supernatant IL-6 at basal condition **(B)** and supernatant TNF-α at polyclonally stimulated condition **(C)**. Bold lines represent median values. Dotted lines correspond to the limit of detection for each cytokine. Mann-Whitney *U*-test was used for comparisons between groups of individuals.

We observed that the presence of circulating EBV in HIV^+^ patients impacted the functional ability of immune cells for responding to a polyclonal stimulus (PMA + ionomycin). In fact, EBV^+^HIV^+^ patients had a significant reduction in the numbers of TNF-α+ CD8^+^ T cells (*p* = 0.017) and multifunctional (TNF-α^+^/IL-2^+^ and TNF-α^+^/IFN-γ^+^) T cells (*p* = 0.046). Notably, IL-6 levels were higher in EBV^+^HIV^+^ patients than those in EBV^−^HIV^+^ patients (*p* = 0.046). This outcome confirms that B-cell chronic stimulation with IL-6 and other proinflammatory cytokines produced during HIV infection could favor the expansion of EBV-infected B-cells (Figures 6A–C).

Lower frequencies of naïve (*p* = 0.022) and central memory (*p* = 0.034) CD4^+^ T cells were found in HIV^+^ patients with AIDS-associated infections than in those who did not present them; as previously reported, this result was found in HIV^+^ patients at stage 3 and with lymphoma. (Figure S3A). Moreover, after the *in vitro* EBV-activation, a reduction of the CD4^+^ TN cell subset was observed in patients who indeed had presented AIDS-defining diseases (*p* = 0.030) (Figure S3B).

Additionally, cell functionality in response to an unspecific stimulus decreased in patients with immunological failure (IF), suggesting that the reduced synthesis of TNF-α and IL-2, which was observed in these individuals, could be greatly attributed to depletion of CD4^+^ T cells, primarily due to the HIV infection (Figure S4).

**Figure 7:**
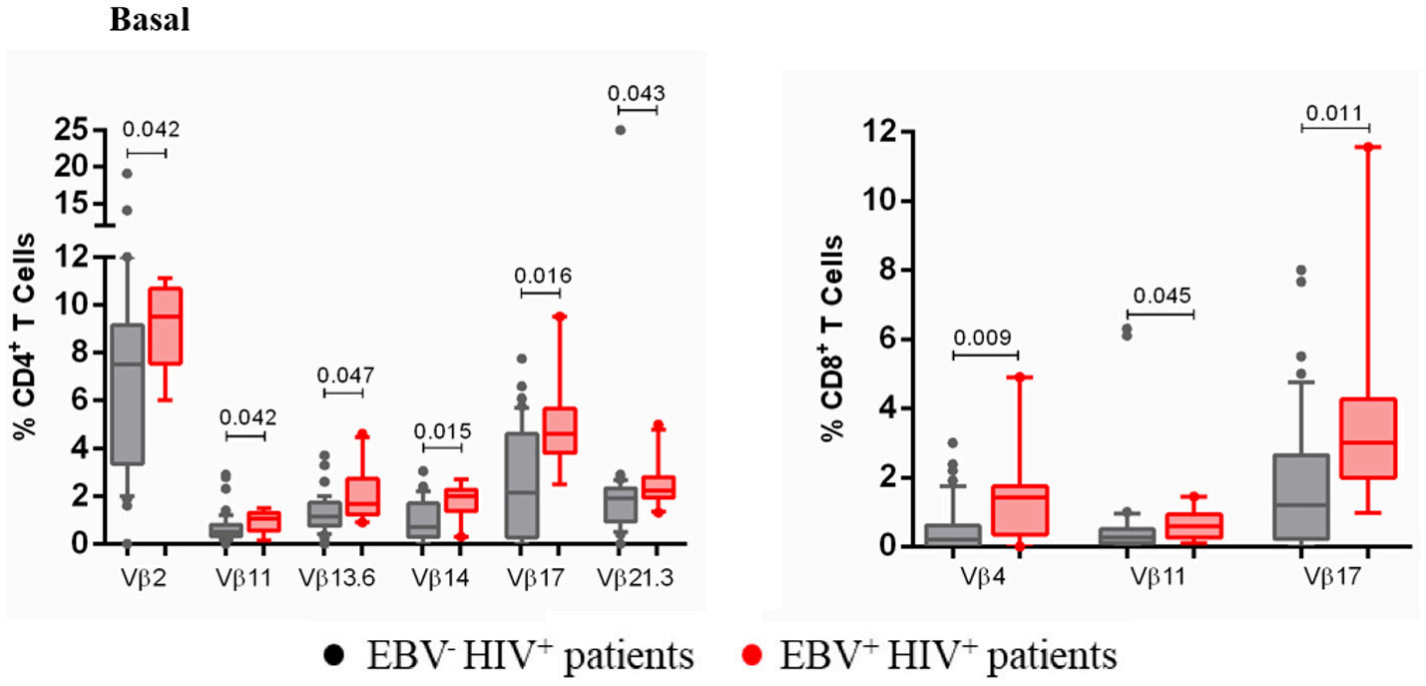
Clonotypic distribution of T cells from EBV^+^HIV^+^ patients. Peripheral blood samples of HIV^+^ patients were cultured *in vitro* at basal (without EBV) conditions. The distribution of CD4^+^ and CD8^+^ T cells positive for some of 24 TCR-Vβ families was analyzed with specific mAbs and flow cytometry. Box and whisker plots show range, median, and interquartile range of percentage of T cells positive for some individual Vβ families. Mann-Whitney *U*-test was used for comparisons between groups.

Lastly, an association was established between EBV antigenic persistence and the altered frequencies of some TCR-Vβ families. In comparison with EBV^−^HIV^+^ patients, EBV^+^HIV^+^ patients had a significant increase in the frequency of Vβ2^+^, Vβ11^+^, Vβ13.6^+^, Vβ14^+^, Vβ17^+^, and Vβ21.3^+^ CD4^+^ T cells, as well as that of Vβ4^+^, Vβ11^+^, and Vβ17^+^ CD8^+^ T cells (*p* < 0.05; Figure 7). Interestingly, the expansion of T cells positive for these families, here observed, suggests that those cells are participating in the EBV-specific response. Indeed, cells positive for these Vβ families increased in number after EBV stimulation in cultures of healthy individuals. This outcome suggests that antigenic persistence provokes functional exhaustion of some activated cell clones, driving them to express death-inducing molecules and causing the deletion of Ag-specific clones.

## Discussion

In this study, EBV-T cell immune responses were evaluated in 56 HIV^+^ patients at different clinical stages of diseases and in 6 HIV^+^ patients newly diagnosed with B-cell NHL who had not received chemotherapy. In addition, plasma EBV load and EBV serology were also evaluated. These tests were carried out to establish any differences in the EBV-related immune response of HIV^+^ patients at different clinical stages and in EBV^+^HIV^+^ individuals. In this last group, the presence of the EBV can play an important role in the development and evolution of B-cell lymphomas.

The results clearly indicated that T cells from HIV^+^ patients at stages 2 and 3, irrespective of the presence of lymphoma, as well as from EBV^+^HIV^+^ patients, have an EBV-specific T cell response of lower quality because they lack mono- and multifunctional CD4^+^ and CD8^+^ T cells. Considering the description of T cells from patients enrolled in the present study, findings correlate with the reduction in T_CM_ and T_EM_ cell subsets in HIV^+^ patients at advanced stages of disease (data not shown).

The Ag-specific T-cell dysfunction observed in patients who we describe here has been reported in chronic diseases, such as those caused by HIV and EBV. In these infections, uncontrolled viremia progressively inactivates T-cell functions, resulting in complete exhaustion (21, 22). Reduction of EBV-specific IL-2^+^ CD4^+^ and CD8^+^ T cells was found in HIV^+^ patients in stages 2, 3, and with lymphoma, which was evident of the first stages of T-cell exhaustion (23, 24). Remarkably, this specific dysfunction was also detected in CD4^+^ T cells from patients in stage 1, suggesting that HIV infection alters the functionality of EBV-specific T cells at early stages and despite ART. Likewise, the presence of EBV in HIV^+^ patients at stage 1 contributes to the first steps of immune exhaustion. It has also been proposed that long-term very high antigenic load could generate a lower number of multifunctional T cells, thus affecting their quality (less control of disease) (22).

During severe stages of immune exhaustion, the production of IFN-γ and TNF-α is eventually compromised (23, 24). This finding was observed in HIV^+^ patients at stages 2 and 3, regardless of the presence of lymphoma, and in CD8^+^ T cells of EBV^+^HIV^+^ patients. This dysfunction was confirmed in the majority of HIV^+^ patients, who produce less IFN-γ in response to EBV. A lower frequency of EBV-specific IFN-γ^+^/TNF-α^+^ CD4^+^ T cells has been described in HIV^+^ patients at early stages of infection and under ART when compared to that of responses to CMV and *Mycobacterium tuberculosis*, which are superior in frequency and multifunctionality (25).

Correspondingly, CD4^+^ and CD8^+^ T cells from HIV^+^ patients with lymphoma were not functional after polyclonal stimulus (PMA + ionomycin; data not shown). Impaired synthesis of IFN-γ and other effector functions of CD8^+^ T cells in response to PMA + ionomycin has been reported in untreated HIV^+^ patients at early stages of infection (26). This result suggests that HIV infection, together with increased cell activation, could functionally block intracellular MAP-ERK1/2-driven responses in CD8^+^ T cells, resulting in a reduction in T-cell proliferation, disruption of differentiation profiles, changes in apoptotic programs and alteration of effector functions (27).

Finally, it has been described that, in the most extreme conditions of functional exhaustion, EBV-specific CD8^+^ T cells can be physically deleted (23, 24). HIV^+^ patients with B-cell lymphoma have shown a loss of EBV-specific mono- and multifunctional CD4^+^ and CD8^+^ T cells. This inadequate EBV immune response probably favored viral reactivation, which is an event that was confirmed by high EBV load in 3 of those patients. The virus could be participating in tumor development and progression in these individuals with a poor prognosis. Similar results in HIV^+^ patients with a high viral load found a decrease in IFN-γ^+^/IL-2^+^ CD4^+^ T cell counts in response to an EBV lysate, together with high expression of activating molecules (28). In conclusion, HIV^+^ patients with lymphoma have a loss of CD4^+^ and CD8^+^ T cells subpopulations that are vital for the response and control of EBV infection.

In addition, circulating levels of IL-6 and IL-10 were detected in sera samples from HIV^+^ and EBV^+^HIV^+^ patients. Particularly, higher levels of IL-6, IL-10, and TNF-α were observed in response to EBV stimulus. Cell sources of IL-6 and IL-10 are diverse and include T cells, monocytes, and B cells (29, 30). In EBV^+^HIV^+^ patients, EBV active infection could induce the synthesis of virus-encoded IL-10 (BCRF1) as well as that of human IL-10 and IL-2 through the action of EBV LMP-1 and LMP-2 proteins that, in turn, could activate the NF-κB pathway (31–33). During HIV chronic infection, the presence of IL-6, IL-10, and TNF-α are associated with poor prognosis and progression to AIDS (2) because they contribute to the chronic activation of B-cells.

Prospective results in HIV^+^ patients have found high serum levels of IL-6 and IL-10 that progressively increased during the years before the diagnosis of AIDS-associated B-cell lymphoma (34). These studies have also reported a relationship between EBV load and high serum levels of IL-6, IL-10, and TNF-α in viremic HIV^+^ patients (35). These observations confirm the hypothesis that HIV contributes to B cell hyperactivation in direct and indirect ways (36), favoring the development EBV-associated hematological malignant tumors (34). The persistent and uncontrolled stimulation of B cells could favor EBV dissemination within them (3, 8, 35) and induce their uncontrolled proliferation by abnormal stimulation of activation-induced cytidine deaminase (AID). This enzyme could contribute to the gain of chromosomal aberrations that affect cell cycle-regulator genes, such as c-MYC; acting together, these mechanisms could lead to the development of aggressive B-cell lymphomas in HIV^+^ patients (8, 36).

This work also evaluated the TCR repertoire diversity with high sensitivity by using a panel of 24 Vβ-specific mAbs that recognize approximately 70% of the Vβ repertoire of CD3^+^ T cells present in healthy individuals. This strategy allowed us to assess the frequency of T cells expressing individual Vβ families (37) and provides a full picture of a large fraction of the T-cell compartment (38).

At basal conditions (without EBV stimulus), all HIV^+^ patients under ART were found to have a lower frequency of CD4^+^ and CD8^+^ T cells positive for the TCR-Vβ families analyzed when compared with those in healthy individuals. Remarkably, individual analyses showed a decrease in the numbers of Vβ2^+^, Vβ8^+^, Vβ17^+^, and Vβ22^+^ CD4^+^ T cells and Vβ2^+^, Vβ7.1^+^, Vβ13.1^+^, Vβ14^+^, and Vβ17^+^ CD8^+^ T cells. It is important to highlight that these families were the most frequently observed in healthy controls, emphasizing their role in the protective response to Ags. In this context, the alterations in some TCR-Vβ families observed in the HIV^+^ patients produce a state of susceptibility and disarray against a variety of pathogenic agents. During chronic HIV^+^ infection, a severely decreased TCR repertoire has been reported, reaching an average of 3-fold fewer different sequences than those in non-infected controls. This exhaustion is consistent with the short-term life of infected CD4^+^ T cells and the significant death of non-infected cells during the progression of disease (6).

The decrease in the number of T cells that are positive for some Vβ families is probably caused by the depletion of HIV-infected cells by the effector function of CD8^+^ T cells or by the accumulation of other antigens (39). Accelerated apoptosis of these cells blocks functional renewal of the TCR repertoire, resulting in a deficient ability of CD8^+^ T cells for controlling viral replication and other infections (39).

Alterations observed here in HIV^+^ patients under ART could suggest that antiretroviral therapy cannot restore the TCR repertoire in individuals whose immune system is already severely impaired by HIV infection. A study confirming this hypothesis showed that individuals with chronic HIV infection were not able to improve the TCR repertoire in CD4^+^ T cells after high active ART (40). After therapy interruption, HIV^+^ patients presented an increase in Vβ10^+^, Vβ14^+^, and Vβ15^+^ CD8^+^ T cell counts and a decrease in Vβ20^+^, Vβ28^+^, and Vβ29^+^ CD8^+^ T cell counts. Nevertheless, these alterations tended to revert after ART was reestablished (39). Recently, HIV^+^ patients were studied before and after ART to evaluate total CD3^+^ TCR repertoire. The results demonstrated expansions of TRBV9 (Vβ1)^+^, TRBV5-6 (Vβ5.2)^+^, TRBV7-6, TRBV7-8, TRBV13 (Vβ23)^+^ and TRBV27 (Vβ14)^+^, as well as reductions in the number of TRBV6-4 and TRBV20-1 (Vβ2)^+^ T cells. In addition, after 3 months under ART, most altered TCR-Vβ families tended to return to their normal values but never reached normality (6). Previously, reported findings lead us to consider that patients studied could have had expansions of TCR families not detected with the panel of mAbs used, given that other alterations were not observed.

Until now, features of the TCR-Vβ repertoire in HIV^+^ patients at different clinical stages or with NHL have not been evaluated after stimulation with a mix of EBV Ags (viral lysate). To our knowledge, this is the first study that describes such responses. Therefore, findings of this study are a valuable and novel contribution.

In the present study, frequencies of TCR-Vβ2^+^, Vβ17^+^, and Vβ22^+^ T cells from all HIV^+^ patients after EBV stimulation were below 30% of representativeness (% of healthy individuals who use a determined family in response to the virus); meanwhile, they were present in >60% of healthy individuals (13, 19). Notably, Vβ7.2^+^ CD4^+^ and CD8^+^ T cells were not detected in patients with lymphoma, indicating the deletion of this family. In addition, some expansions, below 40%, were observed in EBV-specific Vβ23^+^ and Vβ9^+^ CD4^+^ T cells in one patient at stage 2 and another at stage 3, respectively. TCR-Vβ^+^/TNF-α^+^ cells (a more specific marker of EBV response) were not observed in HIV^+^ patients studied due to the disrupted synthesis of the cytokine.

Likewise, the TCR-Vβ repertoire was evaluated in EBV^+^HIV^+^ patients. This group showed a decrease in Vβ3^+^, Vβ9^+^, Vβ14^+^, Vβ17^+^, Vβ18^+^, and Vβ23^+^ CD4^+^ and Vβ3^+^, Vβ9^+^, Vβ17^+^, and Vβ23^+^ CD8^+^ T cell counts when compared to those in healthy individuals. It is likely that reduced frequencies of these T cells leads to altered immune surveillance against EBV, thus favoring its reactivation. It has been proposed previously that continuous and repetitive antigenic stimulation during chronic infections could be responsible for the TCR repertoire narrowing (41) and that increased viral load precedes the generation of lymphomas, as has been observed in immunosuppressed patients (16). Among the TCR families expressed in CD8^+^ T cells mentioned above, Vβ9, Vβ16, Vβ18, and Vβ23 recognize dominant epitopes in BZLF1 and BMLF1 proteins, as well as in LCLs (42, 43); this observation confirms their relevance in the control of EBV infection and possibly in protection against the risk of virus-associated lymphomas. Another alteration observed was expansion, not higher than 20%, of some TCR-Vβ families in the EBV response in this group of patients; for instance, the frequency of Vβ5.3^+^ and Vβ4^+^ CD4^+^ T cells in one patient at stage 1 and in 1 patient with lymphoma, respectively. The expansion of some TCR-Vβ families in the EBV-stimulated cultures of EBV^+^HIV^+^ patients suggests that these clonotypes are frequently used during active EBV infection, although it does ensure their functionality in the case of viral reactivation because the prolonged antigen persistence leads to functional exhaustion. The TCR repertoire of virus-specific CD8^+^ T cells in healthy individuals is widely diverse and renews on a large scale after re-exposition to Ag, with the loss and appearance of new T-cell clonotypes. It is possible to speculate that this Vβ repertoire renewal is attributable to adaptation of the T-cell repertoire to viral variation and/or to clonal senescence (41).

It would be of great interest to evaluate if depletions and expansions of EBV-specific T cell clones positive for some TCR families observed in HIV^+^ patients remain or not because they could be used as predictive markers of clinical evolution toward virus-associated lymphomas. In addition, the routine study of EBV load in HIV^+^ patients could contribute to prevention of long-term morbidity.

The present findings suggest that HIV patients at advanced stages or EBV^+^HIV^+^ individuals exhibit a quantitatively and qualitatively deficient and less protective response to EBV. These alterations could favor EBV reactivation in B-cell subsets and enhance its ability to transform them into tumor cells, thus elevating the risk of clinical evolution toward B-cell lymphoma. Considering the above, it is relevant to monitor patients with positive viral load for EBV, as well as the evaluation of TCRVb families, to determine whether, in the medium- or long-term, patients have a higher risk of evolution to lymphoma.

## Author contributions

DH did the experiments, analyzed data, and wrote the paper. SV, SG, and CH selected patients to include in the study, imparted informant consent, analyzed data, and collected the clinical and biological data of the patients. ML analyzed data. MH and JS selected patients to include in the study and imparted informant consent. SF designed the research and analyzed data. SQ designed the research, analyzed data, and wrote the paper. All authors read and approved the final manuscript.

### Conflict of interest statement

The authors declare that the research was conducted in the absence of any commercial or financial relationships that could be construed as a potential conflict of interest.

## Supplementary material

Figure S1 Clonotypic distribution of T cells from HIV^+^ patients at different clinical stages at basal conditions. Peripheral blood samples of HIV^+^ patients at different stages of disease and healthy controls were cultured *in vitro* at basal (without EBV) conditions. The distribution of CD4^+^** (A)** and CD8^+^** (B)** T cells positive for any of 24 TCR-Vβ families was analyzed with specific mAbs and flow cytometry. Box and whisker plots show range, median, and interquartile range of percentage of T cells positive to individual Vβ families. Mann-Whitney *U*-test was used for comparisons between groups.

Figure S2 Distribution of CD4^+^ and CD8^+^ T cell subpopulations in HIV^+^ patients with viral failure. Peripheral blood samples of HIV^+^ patients were cultured *in vitro* at basal (without EBV) or EBV-stimulated conditions. T cells were analyzed with specific mAbs and flow cytometry. The distribution of naïve (T_N_) and central memory (T_CM_) CD4^+^ T cells at basal condition **(A)**, and the distribution of naïve (T_N_) CD8^+^ T cells at basal and EBV-stimulated conditions **(B)**. Bold lines represent median values. Mann-Whitney *U-*test was used for comparisons between groups of individuals.

Figure S3 Distribution of CD4^+^ T cell subpopulations of HIV^+^ patients with AIDS-defining diseases. Peripheral blood samples of HIV^+^ patients were cultured *in vitro* at basal (without EBV) or EBV-stimulated conditions. T cells were analyzed with specific mAbs and flow cytometry. The distribution of naïve (T_N_) and central memory (T_CM_) CD4^+^ T cells at basal condition **(A)**, and distribution of naïve (T_N_) CD4^+^ T cells at EBV-stimulated conditions **(B)**. Bold lines represent median values. Mann-Whitney *U*-test was used for comparisons between groups of individuals.

Figure S4 Cytokine response to a polyclonal stimulus in HIV^+^ patients with immunological failure Peripheral blood samples of HIV^+^ patients were cultured *in vitro* with a polyclonal (PMA + ionomycin) stimulus, without BFA, for collecting supernatants and measuring the concentration of soluble cytokines by CBA and flow cytometry. Supernatant TNF-α and IL-2 levels are shown. Bold lines represent median values. Dotted lines correspond to the limit of detection for each cytokine. Mann-Whitney *U*-test was used for comparisons between groups of individuals.
