# Supplementary figures and images for: Loss of T-Cell Multifunctionality and TCR-Vβ Repertoire Against Epstein-Barr Virus Is Associated With Worse Prognosis and Clinical Parameters in HIV+ Patients
Source: Front Immunol. 2018 Oct 4;9:2291. doi: 10.3389/fimmu.2018.02291 (PMC6180205; doi:10.3389/fimmu.2018.02291)

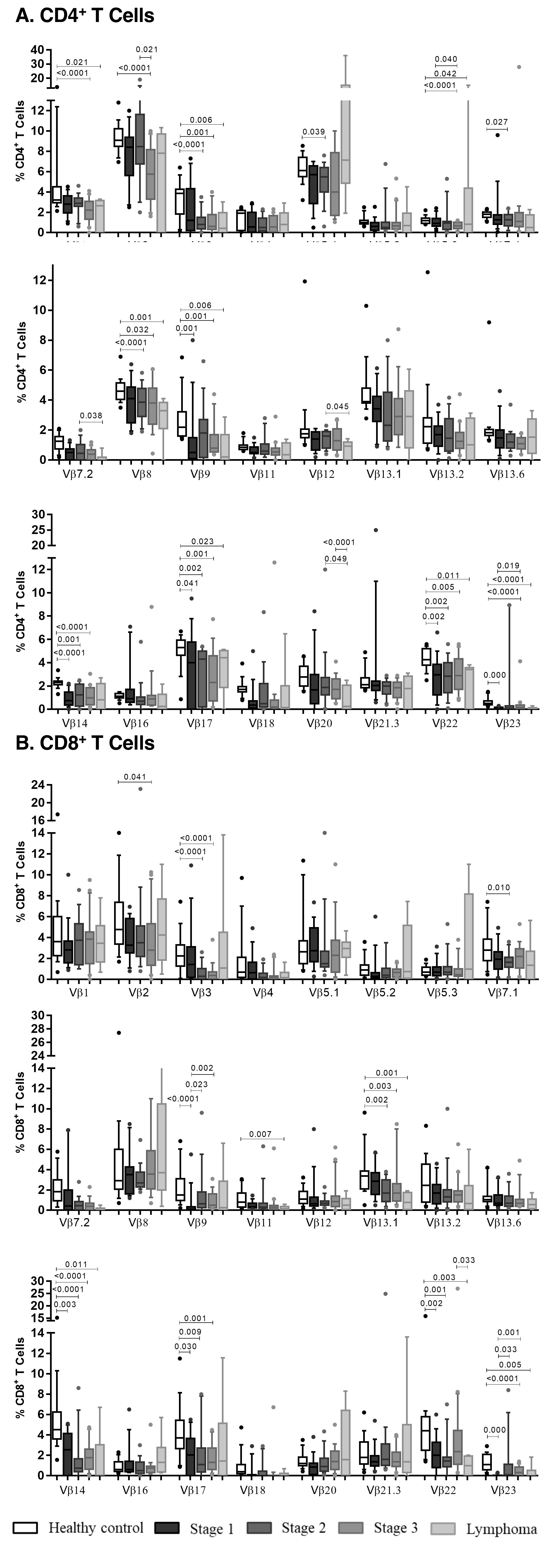

Supplement: Figure S1 — Clonotypic distribution of T cells from HIV+ patients at different clinical stages at basal conditions. Peripheral blood samples of HIV+ patients at different stages of disease and healthy controls were cultured in vitro at basal (without EBV) conditions. The distribution of CD4+ (A) and CD8+ (B) T cells positive for any of 24 TCR-Vβ families was analyzed with specific mAbs and flow cytometry. Box and whisker plots show range, median, and interquartile range of percentage of T cells positive to individual Vβ families. Mann-Whitney U-test was used for comparisons between groups. [file Image_1.JPEG]

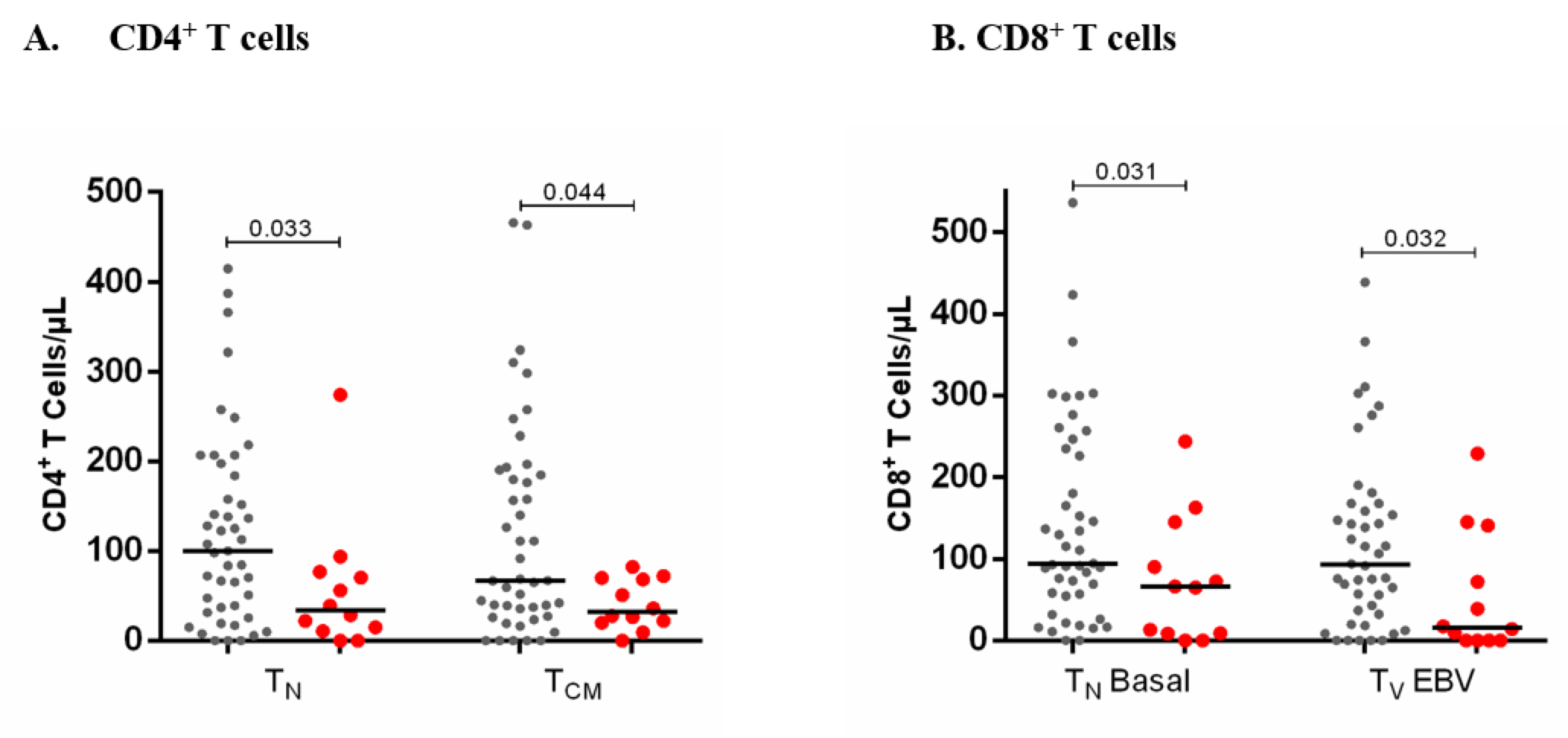

Supplement: Figure S2 — Distribution of CD4+ and CD8+ T cell subpopulations in HIV+ patients with viral failure. Peripheral blood samples of HIV+ patients were cultured in vitro at basal (without EBV) or EBV-stimulated conditions. T cells were analyzed with specific mAbs and flow cytometry. The distribution of naïve (TN) and central memory (TCM) CD4+ T cells at basal condition (A), and the distribution of naïve (TN) CD8+ T cells at basal and EBV-stimulated conditions (B). Bold lines represent median values. Mann-Whitney U-test was used for comparisons between groups of individuals. [file Image_2.JPEG]

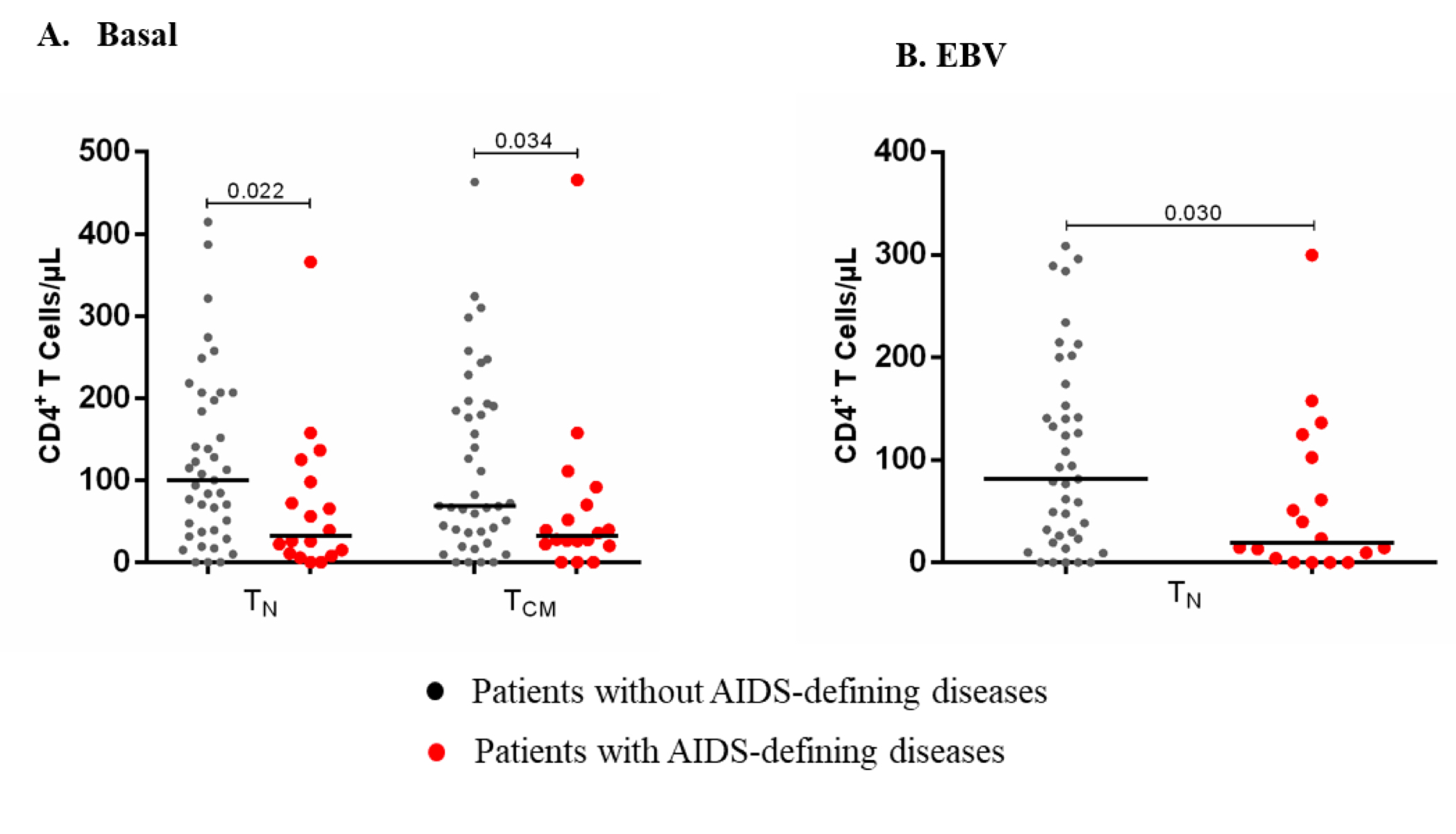

Supplement: Figure S3 — Distribution of CD4+ T cell subpopulations of HIV+ patients with AIDS-defining diseases. Peripheral blood samples of HIV+ patients were cultured in vitro at basal (without EBV) or EBV-stimulated conditions. T cells were analyzed with specific mAbs and flow cytometry. The distribution of naïve (TN) and central memory (TCM) CD4+ T cells at basal condition (A), and distribution of naïve (TN) CD4+ T cells at EBV-stimulated conditions (B). Bold lines represent median values. Mann-Whitney U-test was used for comparisons between groups of individuals. [file Image_3.JPEG]

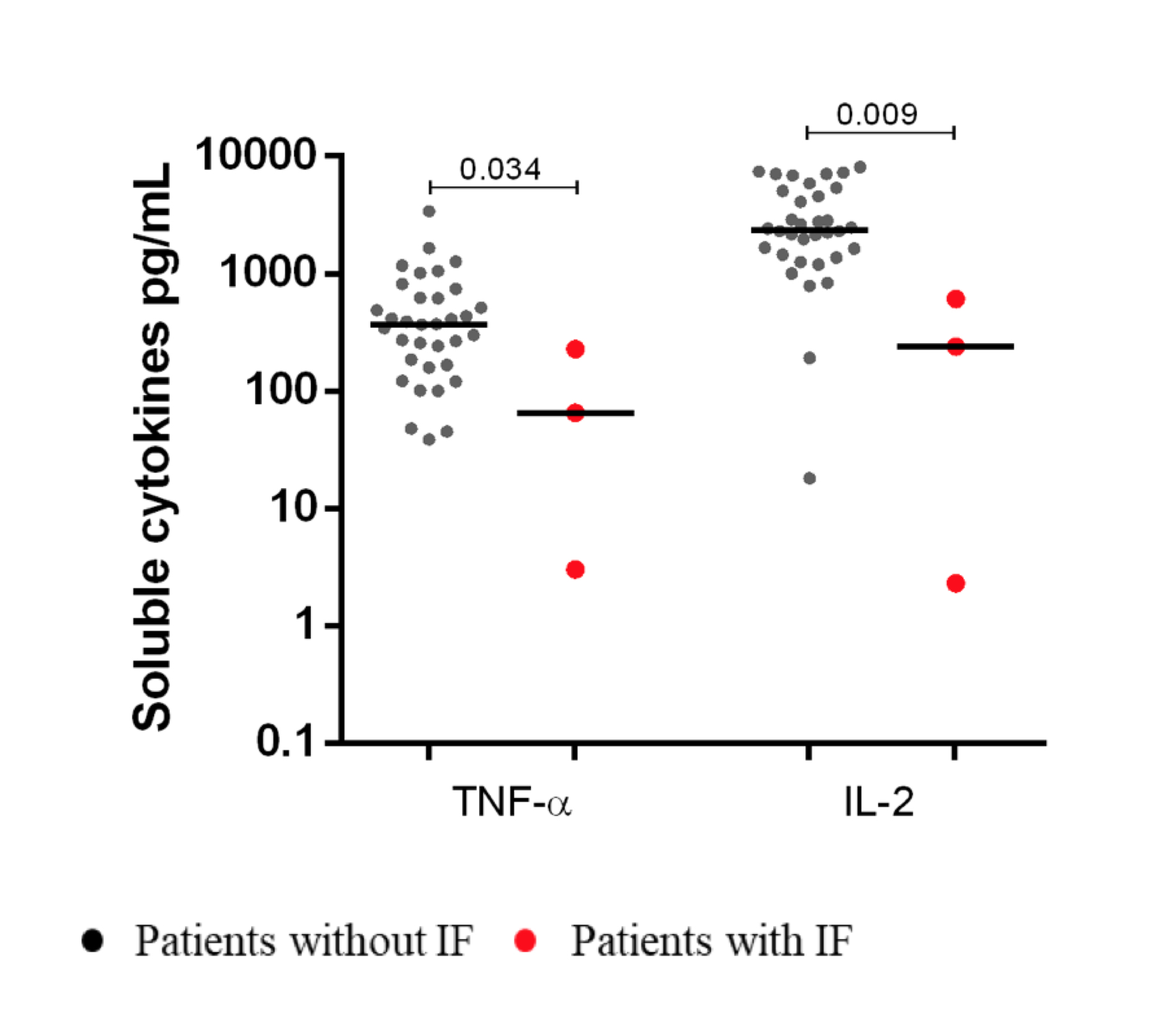

Supplement: Figure S4 — Cytokine response to a polyclonal stimulus in HIV+ patients with immunological failure Peripheral blood samples of HIV+ patients were cultured in vitro with a polyclonal (PMA + ionomycin) stimulus, without BFA, for collecting supernatants and measuring the concentration of soluble cytokines by CBA and flow cytometry. Supernatant TNF-α and IL-2 levels are shown. Bold lines represent median values. Dotted lines correspond to the limit of detection for each cytokine. Mann-Whitney U-test was used for comparisons between groups of individuals. [file Image_4.JPEG]
